# Reported Benefits of Low-Dose Naltrexone Appear to Be Independent of the Endogenous Opioid System Involving Proopiomelanocortin Neurons and β-Endorphin

**DOI:** 10.1523/ENEURO.0087-21.2021

**Published:** 2021-06-15

**Authors:** Marissa J. Metz, Caitlin M. Daimon, Shane T. Hentges

**Affiliations:** Department of Biomedical Sciences and Program in Molecular, Cellular, and Integrative Neurosciences, Colorado State University, Fort Collins, CO 80523

**Keywords:** β-endorphin, hypothalamus, naltrexone, opioid receptor, POMC

## Abstract

Naltrexone is an opioid receptor antagonist approved for the treatment of alcohol and opioid use disorders at doses of 50–150 mg/d. Naltrexone has also been prescribed at much lower doses (3–6 mg/d) for the off-label treatment of inflammation and pain. Currently, a compelling mechanistic explanation for the reported efficacy of low-dose naltrexone (LDN) is lacking and none of the proposed mechanisms can explain patient reports of improved mood and sense of well-being. Here, we examined the possibility that LDN might alter the activity of the endogenous opioid system involving proopiomelanocortin (POMC) neurons in the arcuate nucleus of the hypothalamus (ARH) in male and female mice. Known actions of POMC neurons could account for changes in pain perception and mood. However, using electrophysiologic, imaging and peptide measurement approaches, we found no evidence for such a mechanism. LDN did not change the sensitivity of opioid receptors regulating POMC neurons, the production of the β-endorphin precursor *Pomc* mRNA, nor the release of β-endorphin into plasma. Spontaneous postsynaptic currents (sPSCs) onto POMC neurons were slightly decreased after LDN treatment and GCaMP fluorescent signal, a proxy for intracellular calcium levels, was slightly increased. However, LDN treatment did not appear to change POMC neuron firing rate, resting membrane potential, nor action potential threshold. Therefore, LDN appears to have only slight effects on POMC neurons that do not translate to changes in intrinsic excitability or baseline electrical activity and mechanisms beyond POMC neurons and altered opioid receptor sensitivity should continue to be explored.

## Significance Statement

Naltrexone blocks opioid receptor activity and is used for the treatment of alcohol and opioid use disorders but is also prescribed at lower doses to treat inflammation and pain. A compelling mechanistic explanation for the reported efficacy of low-dose naltrexone (LDN) is lacking, and understanding the central effects of LDN is important, both for basic science and to inform future applications of LDN for central disorders. We hypothesized that LDN might alter the activity of endogenous opioid systems in proopiomelanocortin (POMC) neurons of the hypothalamus. However, we found no evidence for such a mechanism and LDN appears to only slightly affect POMC neurons. We conclude that future studies should shift focus to other opioid systems outside of POMC neurons.

## Introduction

Naltrexone is an antagonist of opioid receptors that has high binding affinity for the μ-opioid receptor (MOR), although it also binds δ- and κ-opioid receptors (Raynor et al., 1994). Naltrexone was approved for use at doses of 50–150 mg to lessen relapse to alcohol and opioid use, which is efficacious largely because of blocking the rewarding actions of these drugs heavily mediated through MORs (Gold et al., 1982; Matthes et al., 1996). In addition to use in treatment for substance use disorders, naltrexone has been prescribed at a much lower dose (3–6 mg) for off-label use in immune-related pain disorders and cancer (Toljan and Vrooman, 2018; Trofimovitch and Baumrucker, 2019). The reported efficacy for low-dose naltrexone (LDN) is paradoxical; MOR agonists, not antagonists, convey analgesic and rewarding properties that can be blocked by application of antagonists. Nonetheless, many anecdotal reports (Ramanathan et al., 2012; Chopra and Cooper, 2013; Ghai et al., 2014; Leonard et al., 2017; Bolton et al., 2020; Zappaterra et al., 2020), *post hoc* studies (Ludwig et al., 2016; Raknes and Småbrekke, 2017, 2019; Raknes et al., 2018), and limited clinical trials (Younger and Mackey, 2009; Younger et al., 2013; Brewer et al., 2018; Lie et al., 2018) suggest that LDN may be useful for treating chronic pain and inflammation. Further, in many of these studies patients report effects such as improved feelings of well-being and vivid dreams (Younger and Mackey, 2009; Younger et al., 2013; Brewer et al., 2018; Lie et al., 2018; Bolton et al., 2020; Zappaterra et al., 2020), and recently LDN has been tested as an adjunct therapy for patients living with depression (Mischoulon et al., 2017) with some promising preliminary results. In the context of naltrexone’s antagonist functions, these benefits are also surprising, as opioid receptor agonism typically induces feelings of euphoria and well-being.

To date, the beneficial effects of LDN have been primarily attributed to inhibition of peripheral inflammatory responses mediated through the Toll-like receptor 4, although a mechanism whereby ultra-LDN (<1 μg/d) acts on a MOR scaffolding protein, filamin A, has also been proposed (Wang and Burns, 2009; Burns and Wang, 2010). Patients receiving LDN often report subjective benefits in the absence of clear objective measures indicative of improvement (Patten et al., 2018). It may be that unrecognized or underappreciated central actions of LDN underpin these anecdotal reports of “feeling better.” Studies from decades ago indicated that LDN could decrease tumor growth in mice, and the authors suggested a mechanism whereby LDN causes a resetting of the endogenous opioid system that allows for a period of rest and re-sensitization of receptors as well as a refilling of endogenous opioid stores to allow the system to function optimally (Zagon and McLaughlin, 1983). Here, we hypothesized that such a mechanism could underlie the positive effects of LDN on subjective affect.

The central β-endorphin system is a key endogenous opioid system within the brain, and knock-out of β-endorphin causes deficits in reward-related behaviors (Hayward et al., 2002) and analgesia (Parikh et al., 2011; Labuz et al., 2016). β-Endorphin is produced in the brain from the precursor peptide proopiomelanocortin (POMC), primarily in neurons in the arcuate nucleus of the hypothalamus (ARH; Veening et al., 2012). Further, POMC neurons of the ARH are heavily regulated both presynaptically and postsynaptically by MORs (Pennock and Hentges, 2011; Fox and Hentges, 2017). Because most of the neurons in the brain that produce β-endorphin are in the ARH, we hypothesized that LDN may exert effects directly on MORs affecting ARH POMC neuron activity and alter the production and release of β-endorphin to partially explain the centrally-mediated actions of LDN.

## Materials and Methods

### Animals

Mice were maintained with approval by the Animal Care and Use Committee of Colorado State University and in accordance with the *Guide for the Care and Use of Laboratory Animals* (Council, 2011). Mice backcrossed to the C57BL/6 strain (The Jackson Laboratory), were group-housed under a 12/12 h light/dark cycle [zeitgeber time (ZT)0 = 6 A.M.] and given *ad libitum* access to food and water. Male and female mice were used for all experiments, and all mice were between 8 and 12 weeks of age at the time of tissue collection. Transgenic mice expressing Discosoma red (*Pomc-DsRed*, gifted by Malcolm Low, University of Michigan) or enhanced green fluorescent protein (*Pomc-eGFP*, Jax stock #009593) under the control of the *Pomc* promoter were used to identify POMC cells during electrophysiological recordings. Mice expressing CRE recombinase driven by the Pomc promoter (*Pomc*^Cre/+^; The Jackson Laboratory, stock #005965) mice were used for stereotaxic injection of the AAVGCaMP6f vector and subsequent GCaMP fluorescence imaging experiments.

### LDN treatment

Naltrexone was dissolved in sterile saline to a concentration of 0.025 mg/ml to make LDN and was stored at −20°C for no more than 7 d. Mice were injected intraperitoneally with 0.1 mg/kg LDN between 7 and 8 A.M. and returned to their home cage. On day 3, 7, or 12, mice were euthanized 2 h after the final injection, when brain naltrexone concentrations will have fallen to, at most, 2–3% of peak (Misra et al., 1976).

### Stereotaxic microinjection

For stereotaxic microinjections, *Pomc*^Cre/+^ animals were first deeply anesthetized with 5% isofluorane and then anesthesia was maintained with 2% isofluorane. Hair was removed and skin was cleaned for surgery before animals were placed in a stereotaxic headframe (David Kopf Instruments) on top of a heating pad. A small hole was drilled into the skull, and a Neurosyringe (2 μl; Hamilton) was lowered into place at 1 mm/s into the ARH (from bregma, A/P: −1.63, M/L: ±0.32, D/V: −6.00). A double-floxed AAVGCaMP6f (AAV9.CAG.Flex.GCaMP6f.WRPE.SV40; Penn Vector Core, University of Pennsylvania School of Medicine, Philadelphia, PA) was injected at 100 nl/min for a total of 200 nl. The needle was left in place postinjection for 10 min and then raised at 1 mm/min. This process was repeated to achieve bilateral injection. During recovery, animals were maintained on a heating pad for at least 1 h and 5 mg/kg carprofen (Rimadyl, Pfizer) was administered daily for 3 d following surgery. LDN injections commenced on day 4 postsurgery and continued for 7 d before GCaMP imaging was performed.

### Patch clamp electrophysiology

On the day of recording between 9 and 9:45 A.M., mice were deeply anesthetized with isoflurane and brains were immediately collected into ice-cold artificial CSF (aCSF; 126 mm NaCl, 2.5 mm KCl, 1.2 mm MgCl_2_•6H2O, 2.4 mm CaCl_2_•2H2O, 1.2 mm NaH_2_PO_4_, 11.1 mm glucose, and 21.4 mm NaHCO_3_) buffered with 95% O_2_ and 5% CO_2_ (carboxygen). Brains were transferred to a Leica VT1200S vibratome also containing ice-cold carboxygen-buffered aCSF and sliced 240 μm thick in the region of the ARH. Slices were then transferred to aCSF kept at 37°C in a water bath. Slices were allowed to rest for at least 1 h before recording.

For recording, slices were maintained in a chamber constantly perfused with carboxygen-infused aCSF kept at 37°C via an in-line temperature controller. For current clamp experiments and experiments isolating spontaneous IPSCs (sIPSCs), MK 801 (15 μm) was added to the bath solution to block NMDA receptors. POMC cells were identified by the presence of the eGFP or dsred reporter visualized through a 40× water-immersion objective (Olympus). Glass patch-pipets were pulled with a Narishige PC-10 vertical pipette puller (Narishige International) to a resistance between 1.4–2.0 mΩ when filled with potassium methyl-sulfate/potassium chloride recording solution (57.5 mm KCl, 57.5 mm CH_3_KO_4_S, 20 mm NaCl, 1.5 mm MgCl_2_, 5 mm HEPES potassium salt, 0.1 mm EGTA, 2 mm ATP, 0.5 mm GTP, and 10 mm phosphocreatine; pH 7.3) for voltage clamp experiments or potassium gluconate recording solution (110 mm K-gluconate, 10 mm KCl, 15 mm NaCl, 1.5 mm MgCl_2_, 10 mm HEPES-potassium salt, 1 mm EGTA, 2 mm Mg-ATP, and 0.2 mm Na-GTP) for current clamp experiments, made fresh weekly and stored at −20°C. An AxoPatch 200B amplifier (Molecular Devices) was used to maintain membrane potential at −60 mV. Test pulses of 10 pA were used to ensure that cells were maintained with a series resistance no greater than 20 mΩ, and that the series resistance did not deviate by >5 mΩ from the original measured value on break in. Recordings were collected via AxoGraph software at 10 kHz and filtered at 5 kHz. [Met5]-enkephalin (ME; Sigma M6638) was prepared as a 10 mm solution in sterile water and kept at 4°C for no more than one month. Before adding to slices, ME was diluted in aCSF, buffered with carboxygen, and passively perfused over the slice during recording. A similar paradigm was followed for preparing drugs for experiments isolating sIPSCs and sEPSCs, where 10 μm of the competitive AMPA antagonist DNQX (Sigma) was perfused onto the slice to isolate sIPSCs, or 10 μm of the GABA_A_ antagonist Bicuculline methiodide (Tocris) was perfused to isolate sEPSCs.

To analyze spontaneous activity, a model sPSC was created from an averaged sample of recordings via Axograph and used as a template for detection. Potassium currents were measured by sampling an average current before ME application and subtracting this value from the peak current measured 2–4 min after drug application.

For action potential threshold experiments, a >1-GΩ seal was obtained in voltage clamp mode and the holding voltage brought to −60 mV. Upon break-in, the AxoPatch 200B amplifier was quickly switched to current clamp mode (I = 0) and the membrane potential recorded. DNQX (10 μm) and Bicuculline (10 μm) were washed onto the slice before and during the experiment to block AMPA-mediated and GABA_A_-mediated currents. After at least 3 m of exposure to these blockers, the cell was current clamped to a membrane potential of –60 mV, and a current ramp of 0–110 pA was applied three separate times.

### GCaMP imaging as an indicator of intracellular calcium levels

For GCaMP fluorescence monitoring, slices were maintained as described above. GCaMP6f was visualized using a 470-nm LED (ThorLabs) and a 40× water immersion objective (Olympus). Slices were allowed to sit for 10 min in the recording chamber before allow the GCaMP6f fluorescence to stabilize in the condition. GCaMP fluorescence was then recorded using CellSens Dimension software (Olympus) at 10 Hz with a 50 ms exposure using an electron-multiplying charge-coupled device (Evolve 512 δ, Photometrics) for 10 min of baseline activity. After 10 min, 10 μm ME was washed onto the slice as recording continued. To analyze baseline GCaMP6f fluorescence, all traces were normalized in Axograph to the reduced level of signal occurring in response to the ME application which significantly decreases calcium influx. These normalized traces were transferred to Prism (version 8) software for area under the curve analysis.

### Perfusion and fluorescent *in situ* hybridization (FISH)

Mice were first deeply anesthetized with sodium pentobarbitol. Transcardial perfusion was then commenced first with 10% sucrose followed by 4% paraformaldehyde, both diluted in potassium phosphate buffer. Brains were extracted into 4% paraformaldehyde solution and stored overnight at 4°C.

On day 1 of FISH, the ARH was collected on ice in 50-μm slices using a Leica VT100S vibratome. Slices were incubated at room temperature in 6% H2O2 for 15 min to quench endogenous peroxidase activity and then incubated for 15 min in proteinase K (10 μg/ml) diluted in PBS containing 0.1% Tween 20 (PBT). Proteinase K was deactivated with incubation in 2 mg/ml glycine in PBT for 10 min. Following two 5-min washes in PBT, tissue was postfixed for 20 min in solution containing 4% paraformaldehyde and 0.2% gluteraldehyde. Tissue was washed once more in PBT, then dehydrated in ascending concentrations of EtOH diluted in DEPC-treated water (50%, 70%, 95%, and 100%) and then briefly rehydrated in PBT. Slices were transferred to vials and prehybridized in 66% deionized formamide, 13% dextran sulfate, 260 mm NaCl, 1.3× Denhardt’s solution, 13 mm Tris-HCl (pH 8.0), and 1.3 mm EDTA (pH 8.0) for 1 h at 60°C. Fluorescein isothiocyanate (FITC)-labeled *Pomc* probe (corresponding to base 532–1000 of GenBank sequence NM_008895.3) was denatured at 85°C for 5 min and then added at 200 pg/μl, along with 0.5 mg/ml tRNA and 10 mm DTT, to the hybridization buffer bathing the slices and allowed to hybridize at 70°C for 18–20 h.

On day 2 following hybridization, slices were first washed at 60°C three times in solution containing 50% formamide and 5× SSC followed by three washes at 60°C in 50% formamide and 2× SSC. Slices were then digested for 30 min at 37°C with RNase A [20 μg/ml in 0.5 m NaCl, 10 mm Tris-HCl (pH 8.0), and 1 mm EDTA] and washed three times for 15 min at room temperature in TNT [0.1 m Tris-HCl (pH 7.5), 0.15 m NaCl, and 0.05% Tween 20]. Slices were blocked for 1 h in TNB (TNT plus 0.5% blocking reagent provided in the TSA kit; PerkinElmer) and then incubated overnight at 4°C in sheep anti-FITC (1:1000; Roche Applied Sciences) antibody conjugated to horseradish peroxidase.

On day 3, probe was detected using a TSA PLUS DNP (HRP) system (PerkinElmer). Slices were then washed for 15 min three times in TNT and then incubated for 30 min in a 1:50 dilution of DNP Amplification reagent. Slices were then washed in TNT and exposed to 1:400 rabbit anti-DNP-KLH conjugated to Alexa Fluor 488 (1 h; Invitrogen) in TNT. Tissue was mounted in Aqua Poly/Mount (Polysciences).

### FISH imaging and analysis

Images were collected using an LSM 800 Airyscan confocal microscope (Zeiss) using Zen Blue software (Zeiss). Stacks of images (six to eight slices per stack) were collected for each slice at an interval of 3 μm through the entire rostral-caudal extent of the arcuate nucleus. All images were collected with the same laser power and digital gain. All analyses were performed using Fiji (ImageJ) software. To control for probe penetration, top and bottom stack images were eliminated before stack merging via max intensity projection followed by intensity-based thresholding. This created a mask for analysis, which then allowed fluorescence intensity of each cell within the stack to be measured. Fluorescence intensity values were subtracted by a representative sampling of the background intensity to control for variability of staining between slices. An average intensity was calculated for each brain from the fluorescent intensities of all analyzed cells.

### Radioimmunoassay

At time of tissue collection, serum samples were collected from trunk blood and stored at −80°C until measurements were made. β-Endorphin levels in plasma were determined using a commercial radioimmunoassay kit according to the manufacturer’s instructions (RK-022-33, Phoenix Pharmaceuticals). In brief, samples were incubated overnight at 4°C with rabbit anti-β-endorphin, followed by another overnight incubation with ^125^I-β-endorphin. Samples were then incubated with goat-anti-rabbit IgG serum and normal rabbit serum, centrifuged, and the supernatant discarded before detection of bound ^125^I-β-endorphin in the remaining pellet with a γ-counter (PerkinElmer). A standard curve was generated from which the concentration of β-endorphin present in each sample was extrapolated.

### Statistics

Results for male and female mice were examined separately for each experiment and were not statistically different. Therefore, all datasets pooled to include both male and female mice. Normality tests were performed on all datasets using the Royston (Royston 1995) method of the Shapiro–Wilk normality test. Normal datasets were analyzed using unpaired *t* tests, and non-normal datasets were analyzed using Mann–Whitney *t* tests where indicated. ME-induced dose-response curves were compared by nonlinear regression and sum-of-squares *F* test to compare EC_50_. All data are presented as mean ± SD. Dose × time responses to LDN were analyzed by two-way ANOVA with Tukey’s multiple comparison test performed in the case of a statistically significant interaction.

## Results

### Treatment with LDN does not alter ME-induced outward currents

**Figure 1.**
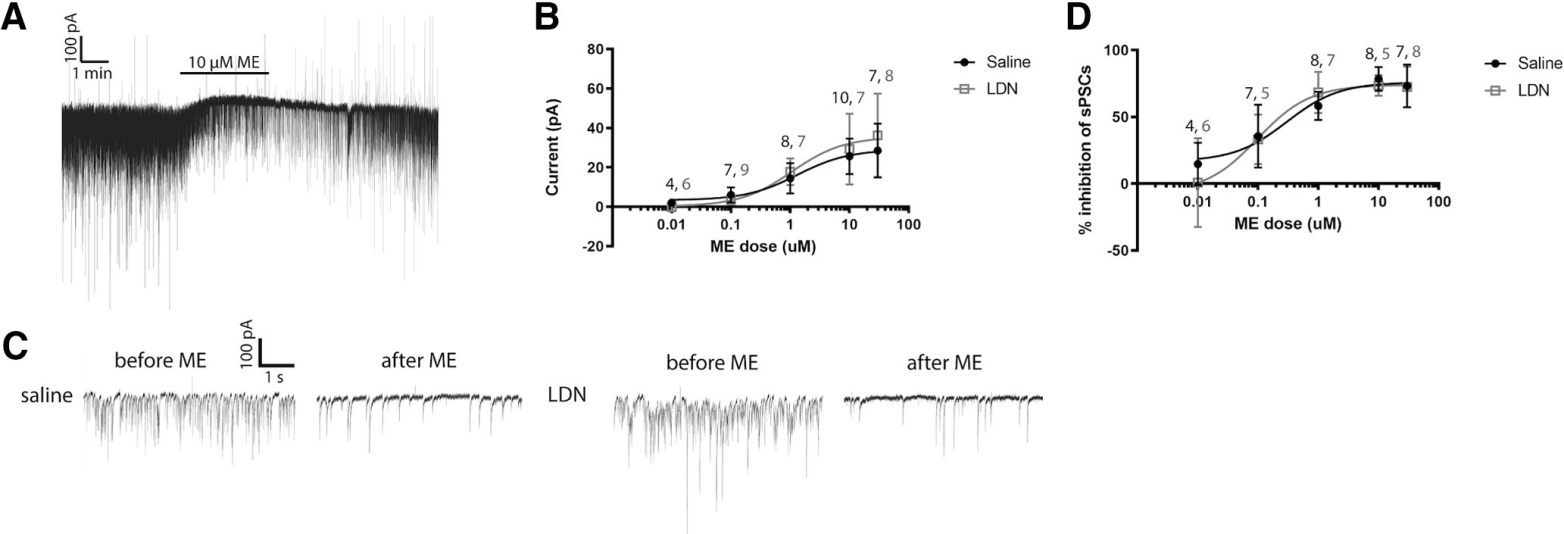
Treatment with LDN did not alter dose responses of opioid receptors on POMC neurons or on neurons presynaptic to POMC neurons. ***A***, Example outward currents elicited by ME application (10 μm). ***B***, Dose-response curves for outward current generated by application of ME. No significant difference was found between dose-response curves. ***C***, Example sPSCs recorded before and after ME application for saline and LDN injected groups. ***D***, Dose-response curves generated from the inhibition of spontaneous events after ME application compared with before ME application in saline and LDN groups. No significant difference was found between dose-response curves. Numbers in black = n for saline and numbers in gray = n for LDN represent the number of cells recorded from for each condition. Slice numbers are the same as cell numbers. Total mice for all dose responses: saline = 21, LDN = 19.

To determine whether LDN might produce its beneficial effects by increasing the sensitivity of opioid receptors on POMC neurons, we examined responses to the opioid receptor agonist ME in slices from mice treated with LDN or vehicle. Mice were injected daily with either 0.1 mg/kg naltrexone in 0.1 ml saline or 0.1 ml saline intraperitonially for one week. This treatment plan was selected as animal studies suggest that this dosage and time period is enough to elicit peripheral effects of LDN (Van Bockstaele et al., 2006; Hammer et al., 2016; Ludwig et al., 2017). On day 7, 2 h after the final LDN injection, brains were extracted and whole cell patch-clamp recordings were made from POMC neurons in slices containing the ARH. ME application induced an outward current, which on POMC neurons has been shown to be mediated largely by MORs coupled to inwardly rectifying potassium channels (GIRKs; Fig. 1*A*; Pennock and Hentges, 2011; Fox and Hentges, 2017). If LDN conferred an increased sensitivity to opioids, ME application would be expected to increase the amplitude of the outward current. However, treatment with LDN did not alter the dose response of the ME-induced peak outward current (EC_50_ saline = 1.315, EC_50_ LDN = 1.071, *F*_(1,67)_ = 0.033, *p *=* *0.855; Fig. 1*B*). Therefore, LDN does not appear to confer enhanced sensitivity or coupling of opioid receptors on POMC neurons to GIRKs.

### Treatment with LDN does not alter ME-induced inhibition of sPSCs

sPSCs, mediated by inputs to POMC neurons, are inhibited by the activation of opioid receptors on presynaptic terminals and show a greater sensitivity to opioid agonist application than outward currents (Pennock and Hentges, 2011). Therefore, we also determined how sPSCs were affected by ME application in LDN-treated and saline-treated mice. Because opioid agonists do not appear to preferentially suppress inhibitory or excitatory inputs (Pennock and Hentges, 2011), both EPSCs and IPSCs were examined at once. As shown in Figure 1 *C*,*D*, no significant change was observed between LDN-treated and saline-treated groups in the inhibition of sPSCs onto POMC neurons in response to ME application (EC_50_ saline = 0.30, EC_50_ LDN = 0.10; *F*_(1,59)_ = 1.219, *p *=* *0.274). Therefore, LDN treatment does not appear to alter the sensitivity of opioid receptor inhibition of transmitter release in neurons upstream of POMC neurons.

### Expression of POMC mRNA and release of β-endorphin peptide

**Figure 2.**
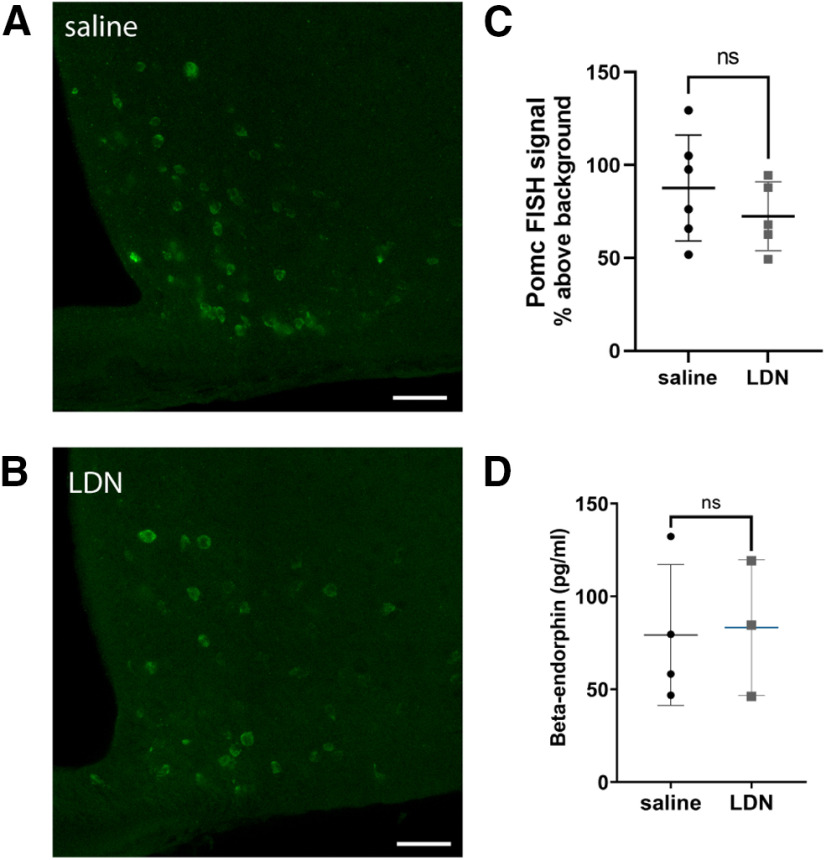
Treatment with LDN did not alter mRNA production of *Pomc* in the arcuate nucleus or release of β-endorphin into plasma. ***A***, Representative FISH image from the medial arcuate nucleus in animal treated with saline and (***B***) LDN. Scale bars: 50 μm. ***C***, No significant difference was observed in *Pomc* FISH intensity between animals treated with saline or LDN. Data points are average intensities of all cells in one brain (saline cell count per brain = 1008 ± 314.7, LDN cell count per brain= 578.8 ± 211.5). ***D***, No change in β-endorphin concentrations in blood plasma was observed after LDN treatment. Each data point represents the average of individual cell fluorescence from one mouse. ns, not significant.

Finding no evidence for overt changes in presynaptic or postsynaptic sensitivity of MORs, we explored the possibility that LDN may alter the production or release of endogenous opioids to mediate the reported efficacy of the treatment. Previous studies have shown that high-dose naltrexone treatment can alter production of POMC peptides relevant to analgesia (Markowitz et al., 1992; Panigrahi et al., 2019). To begin to explore whether LDN may alter the release of the opioid β-endorphin from POMC neurons, we first examined the expression of *Pomc* mRNA as this is the transcriptional precursor to the prohormone from which β-endorphin is cleaved. *Pomc* mRNA was detected using fluorescent FISH in tissue from LDN-treated and vehicle-treated animals. Fluorescence intensity of the *Pomc* FISH signal was determined and reported relative to background fluorescence with all tissue processed and imaged under identical conditions (Fig. 2*A–C*). We found no difference in signal intensity in cells from the two treatments (vehicle vs LDN; *t*_(9)_ = 1.023, *p *=* *0.3329).

While *Pomc* FISH provides an indication of relative mRNA production and potential peptide changes centrally, it is possible that release into plasma is selectively altered by LDN treatment. Therefore, we also examined whether LDN treatment altered the presence of β-endorphin in the plasma of treated animals (Fig. 2*D*). However, no difference was observed in plasma concentration of β-endorphin between LDN-treated and saline-treated animals (saline = 79.17 ± 37.88, LDN = 83.24 ± 36.52 pg/ml, *t*_(5)_ = 0.143, *p *=* *0.892). Therefore, LDN treatment does not appear to alter *Pomc* mRNA production in the ARH nor release of β-endorphin into blood plasma.

### Baseline characteristics of POMC neurons in animals treated with LDN or saline

Despite the lack of noted effects thus far in the studies, we could not rule the possibility that LDN might change the activity of POMC neurons and perhaps peptide release centrally. In fact, during baseline recording for opioid dose-response experiments, it was observed that while opioid responses did not differ, some intrinsic properties of POMC neurons were different in tissue from LDN-treated mice. Intrinsic properties and statistics of POMC neurons from animals treated with LDN or saline are shown in Table 1. POMC neurons from animals treated with LDN had a slightly smaller capacitance than POMC neurons from animals treated with saline and exhibited sPSCs with lower amplitudes and frequency than POMC neurons in the saline group. Recordings from a subset of LDN-treated and saline-treated mice confirmed that the frequency of IPSCs (saline = 7.35 ± 4.97, LDN = 5.08 ± 4.20) was higher than the frequency of EPSCs (saline = 1.83 ± 1.48, LDN = 1.37 ± 1.47) in both groups, confirming previous studies showing that the majority of sPCSs in POMC neurons are mediated by presynaptically-released GABA (Pinto et al., 2004; Hentges et al., 2009). Therefore, it may be that LDN decreases inhibitory tone onto POMC neurons.

**Table 1.** Basal properties of POMC neurons in slices from mice treated for 7 d with LDN or saline

	Resting membrane potential (mV)	Membrane capacitance (pF)	Input resistance (MΩ)	sPSC frequency (Hz)	sPSC amplitude (pA)	AP threshold (mV)	AP frequency (Hz)
Saline mean ± SD	−39.44 ± 6.33 *n* = 10	23.65 ± 7.72 *n* = 32	820.8 ± 737.1 *n* = 32	8.789 ± 4.30 *n* = 33	59.86 ± 31.73 *n* = 33	−38.98 ± 5.78 *n* = 11	7.13 ± 4.08 *n* = 9
LDN mean ± SD	−41.39 ± 11.30 *n* = 11	19.38 ± 6.12 *n* = 34	743.7 ± 699.1 *n* = 34	6.185 ± 4.16 *n* = 32	42.82 ± 23.64 *n* = 31	−41.89 ± 6.25 *n* = 12	5.24 ± 5.76 *n* = 11
*p*	0.6355	0.0151	0.6642	0.0160	0.0183	0.2606	0.4190
*t*(df)	0.4818 (19)	2.498 (64)	0.4362 (64)	2.475 (63)	2.424 (62)	1.156 (21)	0.827 (18)

Membrane capacitance, sPSC frequency, and sPSC amplitude were all lower in the LDN group as compared with saline (*p* > 0.05). Values are expressed as mean ± SD; *n* represents cell numbers. Total slices for each group: (columns 2–5) same as cell numbers, (columns 1, 6, 7) saline = 10, LDN = 9. Total mice for each group: (columns 2–5) saline = 21, LDN = 19; (columns 1, 6, 7) saline = 5, LDN = 4.

### GCaMP-derived calcium responses in POMC neurons are altered by ME

**Figure 3.**
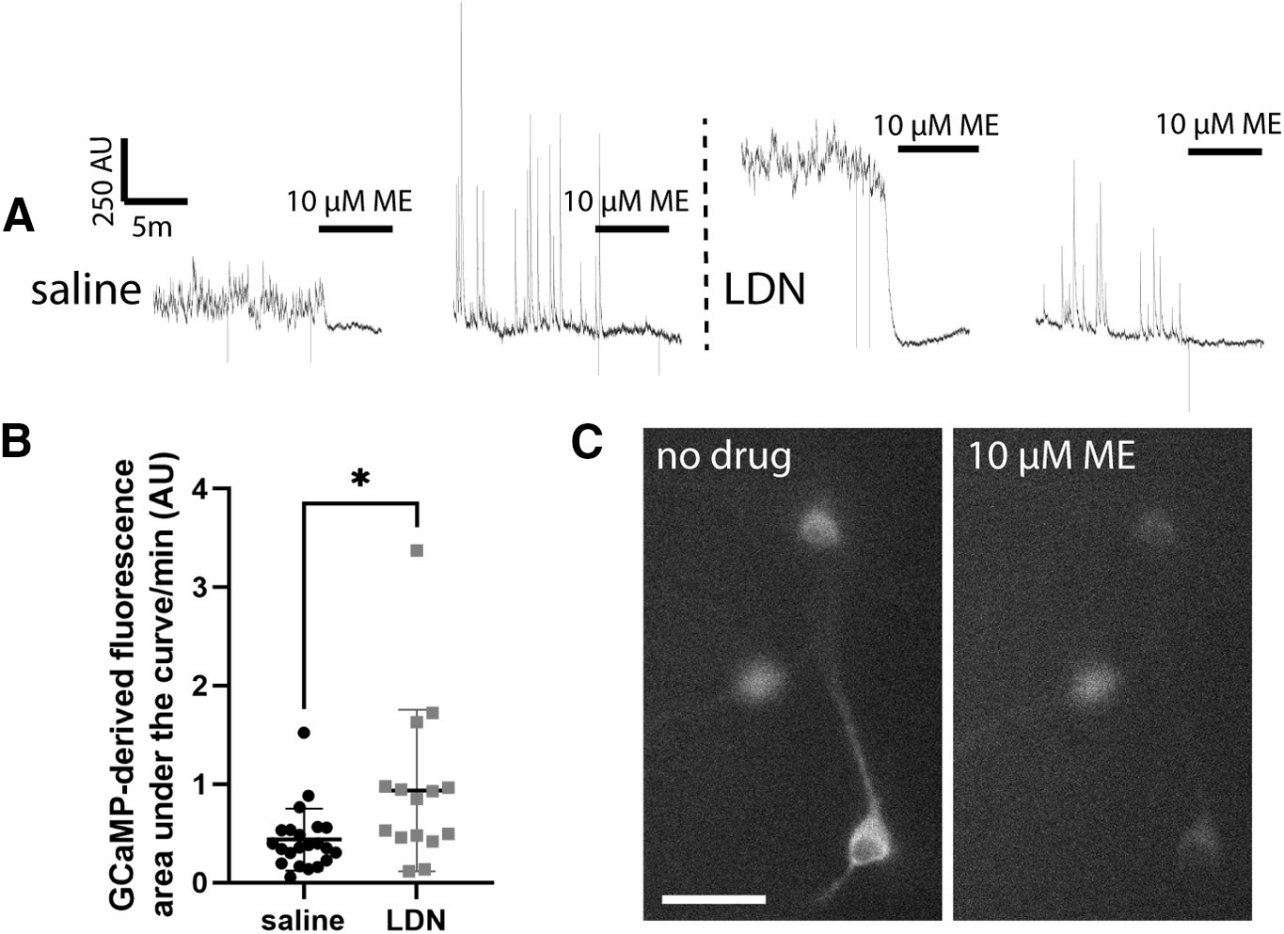
Treatment with LDN slightly increased baseline GCaMP calcium indicator-derived fluorescence. ***A***, Baseline GCaMP-derived fluorescence and fluorescence after 10 μm ME treatment from cells of animals treated with saline or LDN. ***B***, Baseline fluorescence normalized to 10 μm ME application differed significantly between saline and LDN groups; *p < 0.05. Each data point represents one cell. Total slices for each group: saline = 10, LDN = 8. Total mice for each group: saline = 5, LDN = 5. ***C***, Example image of GCaMP expressing POMC neurons before and after treatment with 10 μm ME. Scale bar: 50 μm.

To determine whether the LDN-induced decrease in sPSCs in POMC neurons leads to enhanced POMC neuron activity, we first examined signal indicative of calcium level and flux in POMC neurons from mice treated with LDN or saline as a proxy for POMC neuron activity. GCaMP fluorescence reporter imaging was chosen as a starting point, as this assay has been reported to be a very sensitive indicator of altered POMC neuron activity (Fox and Hentges, 2017) and generally correlates with depolarization and firing rate changes (Jayaraman and Laurent, 2007; Hartung and Gold, 2020). For calcium-dependent GCaMP imaging, AAV containing a floxed sequence for GCaMP6f was delivered into the arcuate nucleus of *Pomc*^Cre/+^ mice and 7 d of LDN administration was completed. GCaMP-derived calcium signal was recorded and normalized to the loss of calcium signal induced by 10 μm ME application after baseline signal recording was completed. This normalization was chosen because of the reliable ME response in cells and because electrophysiological experiments showed no difference in opioid responsiveness between cells in slices from LDN-treated or saline-treated mice. All neurons that appeared healthy and responded to 10 μm ME were included, and baseline calcium fluorescence was analyzed as area under the curve after normalization to ME. POMC neurons from animals treated with LDN showed greater calcium signal at baseline compared with POMC neurons from animals treated with saline (Mann–Whitney *U *=* *1 = 86, *n*_sal_ = 22 *n*_LDN_ = 15, *p = *0.0138; Fig. 3). Therefore, LDN treatment may generally enhance excitability as suggested by the increase in GCaMP-derived calcium signal.

### Intrinsic excitability of POMC neurons

**Figure 4.**
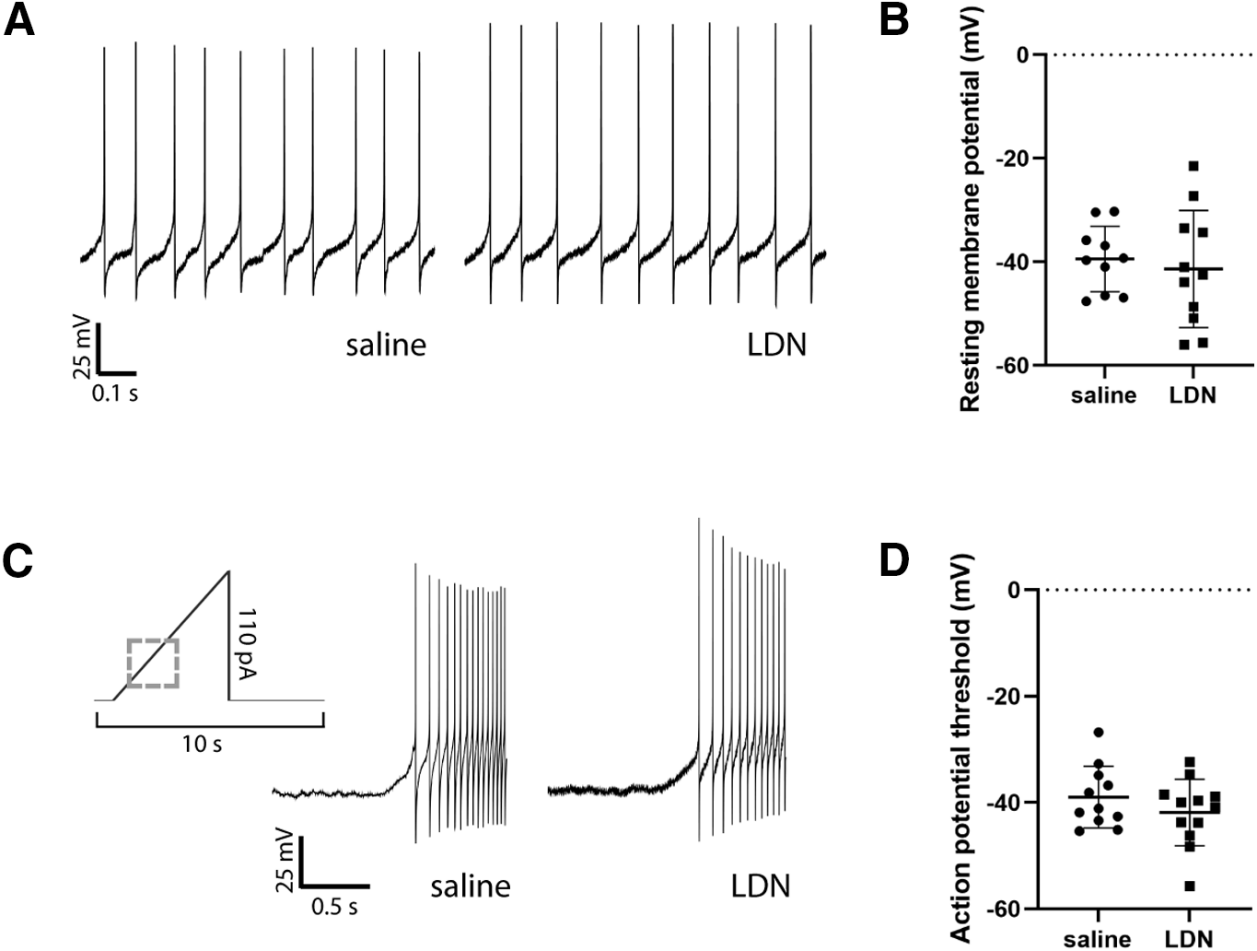
Treatment with LDN did not alter intrinsic excitability of POMC neurons. ***A***, Example current clamp recordings from POMC neurons of saline-treated and LDN-treated mice. ***B***, Resting membrane potential did not differ between POMC neurons from animals treated with saline and LDN. ***C***, Representation of ramp used to determine action potential threshold. The threshold ramp was 5 s and ramped from 0 to 110 pA with a 1-s delay preramp. Dashed gray box represents location of saline and LDN representative traces during action potential ramp. Example traces from saline-treated and LDN-treated cells are shown from the beginning of the ramp protocol, with action potentials beginning 0.3 s after initiation of the ramp. ***D***, Action potential threshold did not differ between POMC neurons from animals treated with saline and LDN. Each data point represents one cell. Total slices used for each group (from a total of saline = 5 and LDN = 4 mice per group): saline = 10, LDN = 9.

To test whether an increase in GCaMP-based calcium activity was indeed accompanied by an increase in POMC neuron intrinsic excitability, action potential thresholds were determined for POMC neurons from animals treated with LDN and saline (see Table 1). Neither resting membrane potential frequency of action potentials was different between groups (Fig. 4). Action potential threshold was measured in the presence of presynaptic blockers for GABA_A_ receptors (Bicuculline methiodide, 10 μm), AMPA receptors (DNQX, 10 μm), and NMDA receptors (MK-801, 15 μm). Action potentials were elicited with a 0- to 110-pA current ramp. The amount of current required to elicit an action potential from a holding potential of −60 mV did not differ between groups (saline = 33.63 ± 21.69 pA, LDN = 30.09 ± 23.86 pA, *t*_(17)_ = 0.3215, *p *=* *0.7518; Fig. 4*C*,*D*). Therefore, LDN treatment does not appear to alter baseline firing nor intrinsic excitability of POMC neurons.

### Dose × time response of POMC neuron intrinsic excitability and β-endorphin release

**Figure 5.**
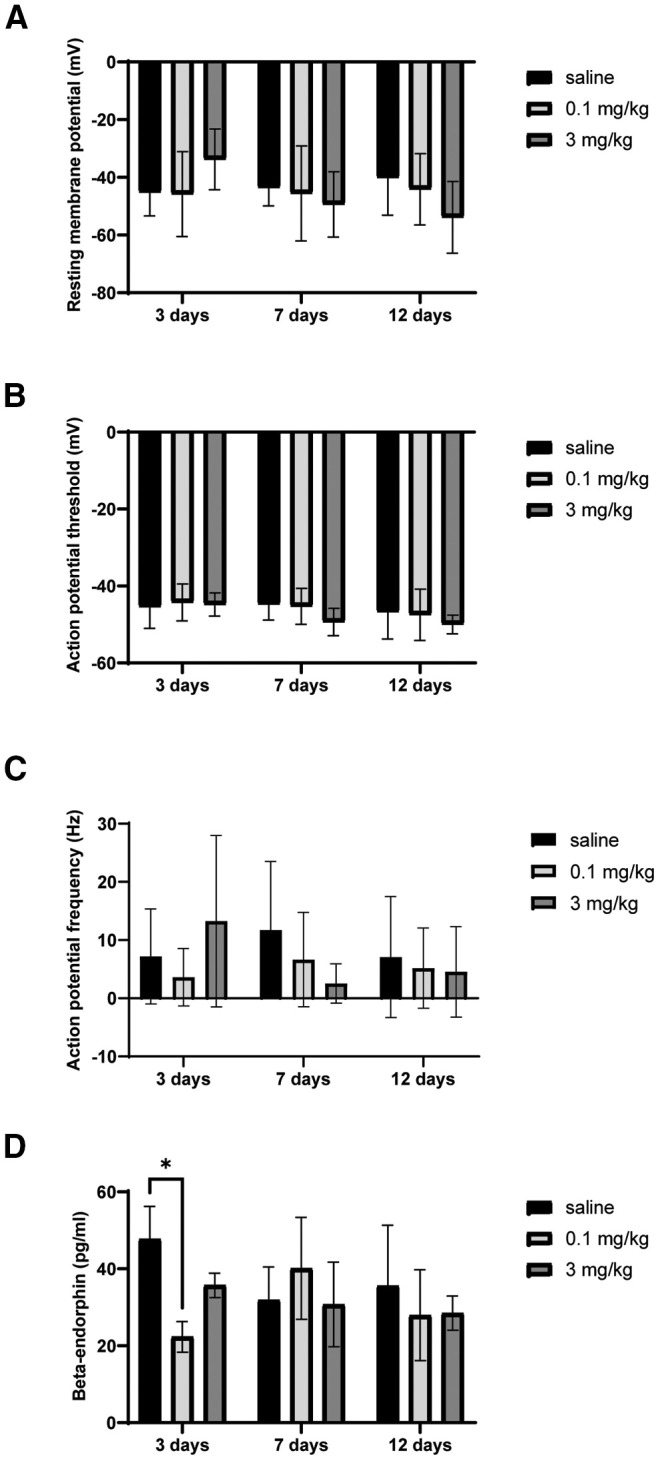
Increased dose of LDN or varied the time of LDN exposure did not cause sustained changes in POMC neuron intrinsic excitability or β-endorphin plasma concentrations. ***A***, Resting membrane potential did not change after 3-, 7-, or 12-d LDN treatment at either 0.1 or 3 mg/kg. ***B***, Varying the time or dose of LDN treatment also did not change POMC neuron action potential threshold or (***C***) action potential frequency. ***D***, LDN treatment did not systematically alter β-endorphin concentrations in plasma, although a transient decrease in β-endorphin concentration was observed with 0.1 mg/kg at 3 d of treatment. Number of mice used for each group in the β-endorphin group are as follows: 3 d saline = 3, 0.1 mg/kg LDN = 3, 3 mg/kg LDN = 4; 7 d saline = 7, 7 d 0.1 mg/kg = 8, 7 d 3 mg/kg = 4; 12 d saline = 5, 12 d 0.1 mg/kg LDN = 4, 12 d 3 mg/kg = 4; *p < 0.05.

While the above experiments indicate that 7 d of 0.1 mg/kg LDN does not appear to change β-endorphin release into plasma or POMC neuron intrinsic excitability, it is possible that LDN effects on the β-endorphin system could be short-lasting or take longer than 7 d to manifest. Therefore, we examined these parameters after 3, 7, and 12 d of LDN exposure at 0.1 and 3 mg/kg LDN compared with saline controls (Table 2). No main effects of dose (*F*_(2,73)_ = 0.39, *p *=* *0.68), nor time (*F*_(2,73)_ = 1.31, *p *=* *0.28) were apparent for resting membrane potential (Fig. 5*A*). Similarly, action potential threshold did not appear to be altered by dose (*F*_(2,74)_ = 2.82, *p *=* *0.60) or time (*F*_(2,74)_ = 2.02, *p *=* *0.14; Fig. 5*B*), and action potential frequency also showed no main effects of dose (*F*_(2,73)_ = 0.45, *p *=* *0.64) or time (*F*_(2,73)_ = 0.97, *p *=* *0.38; Fig. 5*C*). β-endorphin concentrations in plasma were also measured after the dose × time experiment and there were no main effects (dose: *F*_(2,32)_ = 0.62, *p *=* *0.54; time: *F*_(2,32)_ = 2.23, *p *=* *0.12), but an interaction was observed between groups (*F*_(4,32)_ = 2.95, *p *=* *0.04) with the concentration of β-endorphin being slightly lower in the 0.1 mg/kg group compared with the saline group at 3 d (*p = *0.02; Fig. 5*D*). No other groups were significantly different in their plasma β-endorphin concentrations within any of the treatment time blocks.

**Table 2.** Baseline properties of POMC neurons from animals treated with varied doses and times of LDN

	RMP (mV)	AP threshold (mV)	AP frequency (Hz)
Saline 3 d mean ± SD	−45.18 ± 8.16 *n* = 12	−45.42 ± 5.58 *n* = 13	7.18 ± 8.15 *n* = 12
1 mg/kg 3 d mean ± SD	−45.82 ± 14.70 *n* = 9	−44.26 ± 4.80 *n* = 8	3.61 ± 4.93 *n* = 9
3 mg/kg 3 d mean ± SD	−33.77 ± 10.52 *n* = 10	−44.81 ± 3.02 *n* = 9	13.26 ± 14.73 *n* = 10
Saline 7 d mean ± SD	−43.60 ± 6.28 *n* = 8	−44.74 ± 4.14 *n* = 8	11.73 ± 11.80 *n* = 8
1 mg/kg 7 d mean ± SD	−45.56 ± 16.49 *n* = 11	−45.29 ± 4.69 *n* = 11	6.65 ± 8.11 *n* = 11
3 mg/kg 7 d mean ± SD	−49.37 ± 11.36 *n* = 8	−49.39 ± 3.54 *n* = 8	2.54 ± 3.39 *n* = 8
Saline 12 d mean ± SD	−40.11 ± 12.99 *n* = 10	−46.71 ± 7.09 *n* = 11	7.08 ± 10.41 *n* = 10
1 mg/kg 12 d mean ± SD	−44.11 ± 12.36 *n* = 6	−47.49 ± 6.66 *n* = 7	5.19 ± 6.90 *n* = 6
3 mg/kg 12 d mean ± SD	−53.86 ± 12.44 *n* = 8	−50.00 ± 2.44 *n* = 8	4.53 ± 7.78 *n* = 8

Resting membrane potential, action potential threshold, and action potential frequency mean ± SD are presented for cells from slices taken from mice treated with saline, 0.1 or 3 mg/kg LDN for 3, 7, or 12 d. No main effects were detected for any parameter. *n* = number of cells recorded from and comes from 2 or more slices from at least three mice.

## Discussion

Overall, LDN appears to have minimal effects on the activity of POMC neurons. The amplitude and frequency of PSCs onto POMC neurons was slightly decreased, and GCaMP-derived calcium signal from POMC neurons was increased. However, the resting membrane potential, firing frequency, and intrinsic excitability of POMC neurons were all unaltered by LDN, even when dosage and time of treatment was altered. Consistent with this, LDN treatment did not consistently change the production of *Pomc* mRNA or the release of β-endorphin into blood plasma. Thus, we found no evidence to support the hypothesis that LDN may exert its reported mood enhancing and pain reducing actions by stimulating the endogenous β-endorphin system.

It has repeatedly been shown that high doses of naltrexone increase MOR expression throughout the brain (Tempel et al., 1984; Unterwald et al., 1995, 1998; Díaz et al., 2002). Further, studies examining behavioral effects show enhanced respiratory depression and analgesia after extended high-dose naltrexone followed by acute agonist administration (Díaz et al., 2002); therefore, it appears these upregulated opioid receptors are functional. However, our studies indicate that LDN does not increase coupling to effectors in POMC neurons, and likely does not induce increased expression, unless there are extra non-signaling receptors. Thus, LDN does not appear to have the same effect as higher, more chronic doses of naltrexone *in vivo* on opioid receptors in POMC neurons.

The lack of consequences found in the current study do not rule out that LDN could be affecting opioid receptor function in other brain regions. Opioid receptor expression is widespread throughout the central nervous system (Le Merrer et al., 2009; Corder et al., 2018), and some regions may be more sensitive to antagonist-induced changes than POMC neurons. While long-term analgesic effects of LDN are speculated to occur peripherally, it is known that top-down activation is important for pain relief. The central analgesic system contains the periaqueductal gray (PAG), which sends inhibitory projections to the rostral ventromedial medulla (RVM), and both of these brain regions are known to express opioid receptors, which could be affected by LDN (Gutstein et al., 1998). Further, effects on mood and dreaming reported by many patients taking LDN likely have a central mechanism, and several brain regions involved in affecting mood, including parts of the limbic system, express opioid receptors (George et al., 1994; Zubieta et al., 2003). Thus, LDN could affect opioid receptor systems in these regions to account for alterations to mood previously reported by patients.

Investigation of central β-endorphin expression and release in response to LDN also revealed little change. Changes in endogenous β-endorphin tone after LDN were hypothesized to occur because β-endorphin is known to improve affect and thus could account for improvements in mood reported by patients taking LDN. Further, previous studies administering high-dose naltrexone show increases in β-endorphin and POMC peptide expression in plasma and brain (Nikolarakis et al., 1987; Markowitz et al., 1992; Jaffe et al., 1994; Gordon et al., 2017; Panigrahi et al., 2019). Therefore, we examined the effect of LDN on *Pomc* transcript and β-endorphin in plasma. It is worth commenting on the different *y*-axis scale bars referring to β-endorphin concentrations in Figure 2 versus Figure 5. The samples shown in Figure 5 were analyzed using reagents with different production lot numbers as those used in Figure 2 and this likely explains the difference observed in absolute concentration. Within each experiment, it is clear no difference in plasma β-endorphin levels is observed whether animals receive saline or one of two doses of LDN except that only 0.1 mg/kg LDN treatment for 3 d, may have slightly decreased β-endorphin concentration in plasma. This was in the opposite direction of our hypothesized increase in β-endorphin but could be indicative of an earlier release event followed by a dip in plasma peptide concentration. However, if anything, this change is transient and seems to resolve by 7 d, therefore it is unlikely to explain the reported efficacy of LDN that may last for months or years (Ludwig et al., 2016).

It is also plausible that LDN could have effects on other opioid peptide systems in the brain. POMC neurons of the ARH are the most prominent source of β-endorphin in the brain (Zakarian and Smyth, 1982). However, other opioid peptides, such as enkephalins and dynorphins, are also known to affect anxiety, aversion, and stress, all of which could be related to mood changes reported by patients using LDN (Wittmann et al., 2009; Femenía et al., 2011; Ménard et al., 2013; Melo et al., 2014; Nam et al., 2019). The production of precursors for enkephalin and endorphin are much more widespread in the brain than the production of the precursor polypeptide POMC (Le Merrer et al., 2009; Corder et al., 2018), and thus the ease in detecting changes in these systems would likely depend on how generalized the alterations in the production of those specific peptides are, if present at all. Peripherally, LDN does appear to induce increases in the level of met-enkephalin in patients with multiple sclerosis (Ludwig et al., 2017), therefore changes in central enkephalin systems may also be possible. Further, many previous studies use LDN treatments for populations in which opioid systems may already be dysfunctional, and LDN may be acting to reset this system back to normal. Because our studies were in healthy animals, we cannot rule out the possibility that LDN could confer effects in conditions of pathologic opioid system function.

Overall, our lack of LDN-induced changes within POMC neurons does not rule out changes in animals with dysfunctional opioid tone, changes with longer term LDN treatment, or changes within the rest of the brain. Understanding the central effects of LDN is important, not only from the perspective of basic science, but also to inform future applications of LDN, as LDN is already being explored as a potential treatment for depression (Mischoulon et al., 2017). From our studies, it appears that future inquiries would be best served by focusing on opioid systems other than the central β-endorphin system or by examining the effects of LDN in conditions where dysfunctions in β-endorphin tone likely exist.

## Synthesis

Reviewing Editor: Quentin Pittman, University of Calgary Decisions are customarily a result of the Reviewing Editor and the peer reviewers coming together and discussing their recommendations until a consensus is reached. When revisions are invited, a fact-based synthesis statement explaining their decision and outlining what is needed to prepare a revision will be listed below. The following reviewer(s) agreed to reveal their identity: Richard Bodnar.

The authors examined whether low-dose naltrexone (LDN) exerted its analgesic and increased “well-being” effects observed in humans through an arcuate POMC mechanism in a mouse model. The authors utilized an impressive range of neurophysiological, molecular, biochemical and neuroanatomical approaches in examining this hypothesis. They tested whether LDN might alter the activity of the endogenous opioid system involving hypothalamic arcuate POMC neurons in male and female mice. However, the authors found that LDN failed to change the sensitivity of opioid receptors regulating POMC neurons, the production of the ꞵ-endorphin precursor Pomc mRNA, nor the release of ꞵ-endorphin into plasma. Spontaneous post-synaptic currents onto POMC neurons were slightly decreased after LDN treatment and GCaMP fluorescent signal, a proxy for intracellular calcium levels, was slightly increased. However, LDN treatment did not appear to change POMC neuron firing rate, resting membrane potential, nor action potential threshold.

The reviewers and I agree that the results are clear and of high quality. None the less, we believe that the paper would have greater impact with attention to the following points:

Major:

1. The major conclusion of this study rests on the failure to observe an effect. It is compelling, but needs more evidence. The major shortcoming of the study was its use of one and only one paradigm mimicking in mice the effects of LDN in humans. In all studies, naltrexone was injected intraperitoneally with 0.1 mg/kg of a concentration of 0.025 mg/ml once a day for 7 days and sacrificed 2 h after the final injection. It is critical that the authors provide us with a combined dose-response and “time"-response (different intervals) curve for at least one of the critical variables that failed to elicit a response. If the dose/time response study turns out to produce effects, then they need to pursue it further. If not, it will give the reader greater assurance that the “negative” effects are indeed germane.

Minor:

2. In the introduction, please offer better rationale as to why the POMC neurons are thought to possibly be involved in pain or use disorders. While negative results in themselves are not a problem, it is important that there is a good rationale for considering that there might have been 'positive' results.

3a. sPSC recordings - Given the difference in synaptic event frequency and amplitude, please indicate if the difference is ascribed to both glutamatergic and GABAergic events.

3b. Similarly in Figure 1, please consider whether ME preferentially suppresses one type of events over the other?

3c. As MORs are referenced to mediate the effects of ME, please consider showing that a MOR antagonist would block ME-mediated sPSC inhibition.

3d. As no pharmacological experiments were completed to confirm that the outward current observed is GIRK-mediated, please consider referring to the outward current observed in voltage-clamp recordings as a net “outward current” rather than GIRK current. This should be revised in the methods and legends

4a. It seems that the description of FISH experiments is missing in the Methods. Please also include a description of how the FISH signal was quantified. were the slices matched in saline vs LDN tissue and was the signal intensity quantified in each cell or over the whole area.

4b. In Figure 2, please indicate the number of cells surveyed to make up each data point. Please also add a scale bar and indicate the FISH signal hybridized on the figure panel.

5a. In Figure 4, please consider placing the saline vs LDN traces horizontally so that magnitude of ME-mediated fluorescence change can be better compared.

5b. In panel C, please label the representative cell with an arrow or asterisk so it is clear which cell is being compared.

6. Figure 4 legend - please reword the last sentence so that it is clear the cells represented are from 4-5 mice in total and 1-2 slices per mouse. Currently it reads as if there would be 9-10 slices from each of 4-5 mice.
